# Artificial Crowns

**Published:** 1881-06

**Authors:** Charles J. Siddall


### THE DENTAL REGISTER.

VOL. XXXV.] JUNE, 1881. [No. 6.

### ARTIFICIAL CROWNS.

BY CHARLES J. SIDDALL, D. D. S.

It has long been the practice to adjust artificial crowns upon healthy roots of teeth, the crowns of which have been lost; especially upon the roots of the anterior teeth. The method first employed was simply to attach a porcelain crown by means of a pivot, either of wood or of some suitable metal.

Crowns attached by this method have been, and still are, worn with much comfort, and answer the purpose very well. They are, however, not very firm, and will not resist the force of mastication, except in the most favorable cases. Many methods have been devised with the view of overcoming this difficulty. The method which at present seems to meet with most favor, and which it is the object of this paper to describe, is known as "The Richmond method," from the name of the man who first devised it.

If an operation of this kind is to be attended with the best success, the following considerations are important.

First, the root must be strong, well developed, and free from disease. If the pulp has been destroyed by disease, and the adjacent tissues have been involved in inflammation, the result *may* not be entirely successful; still, even under these circumstances one would be justified in making the attempt.

Second, the peculiar constitutional diathesis of the patient will have a great influence upon the success of the undertaking. Some persons are so constituted that the slightest circumstance will induce difficulties out of all proportion to the cause.

Lastly, but by no means of least importance, the manner of performing the operation. This is an operation requiring not a little skill and considerable knowledge, and may be performed in so bungling a manner as to destroy all hope of success.

Now as to the method of procedure. It is necessary, where a part of the crown is to be made of porcelain, that the tooth be cut off a little below the margin of the gum. If a considerable portion of the crown remain and the pulp be still alive, that must be destroyed, either by means of escharotics, or, what is better, by excision; since it is less likely to be followed by inflammation. In the latter case the canal may be immediately filled, with very little danger of any difficulty. In case the pulp has died the same treatment will be required as with any devitalized tooth.

Meantime, if the inflammation is not too great, a narrow gold band should be accurately fitted about the root, extending from the process to the free margin of the gum. The edge of the band next the process should be made to conform to the shape of the process, and should be dressed to a knife edge, to facilitate its easy passage between the gum and the root. It should fit against the process as accurately as possible, but should not pass between that and the root. The gold best adapted to this purpose may be prepared by rolling a gold coin to about 20 standard gauge, and the solder used is made by adding to coin gold one part in five of fine brass wire.

The next step is the adjustment of a gold tube or wire to the enlarged opening in the root. This pivot should extend as far into the root as possible without endangering the walls of the canal, and must project beyond the root somewhat farther than the band. It should fit loosely, to allow the introduction of some plastic material on the final adjustment.

It is now necessary to take an impression of the parts, including several of the adjoining teeth, with the band and tube in place. On removing the impression the tube will come away with it, and the band must be removed from the root and placed in its proper position in the impression and a model obtained. If the band needs any more dressing it is easily done, either with a file or corundum wheel. On the anterior side the band should not show above the gum.

A platinum cap is now to be fitted inside the band, perforated to receive the tube, which is now to be cut off almost on a level with the surface of the cap. The thing next in order is to select and fit into the space a suitable, plain plate tooth. This should be made to fit accurately, and is to be backed with platinum. The backing should extend *over* the end of the tooth, so as to aid in drawing the solder to the outer edge of the platinum cap, thus filling the whole joint.

When the tooth has been fitted, and firmly waxed in place, as much of the plaster should be cut away as can be done without disturbing the several pieces, and the whole invested for soldering. The posterior portion of the crown is now to be restored with solder. If necessary, small scraps of gold may be added to aid in forming the desired shape. After the piece is soldered it should be finished just as a filling would be, and the whole firmly pressed into place, enough oxy-phosphate of zinc being placed between the crown and root to fill the joint. Any surplus of the cement will escape about the root, and should be carefully removed to avoid irritation.

The method of adjusting crowns to the roots of the posterior teeth is much more simple, and perhaps does not need a detailed description.

These crowns consist simply of a band, wide enough to restore the height of the crown, covered with a cap upon which cusps are built to restore the shape. This is forced on to the root, with enough oxy-phosphate of zinc to fill all the space between the root and the crown. In this way crowns may readily be adapted to teeth in which the pulp is still alive.

In adjusting these crowns it has been found that if the phosphate of zinc is used, any excess of material that may escape about the gum is less likely to cause irritation than if the chloride is used. Some writers recommend that an opening be made in the crown for the escape of any excess, the opening afterward to be filled with foil. This seems unnecessary if care is taken to remove all that escapes about the root. Sometimes platinum is used to form the band, especially in the case of the anterior teeth. It is much more easily soldered, and the color is certainly no more objectionable than that of gold.

